# Effects of desensitizing dentifrices on dentin tubule occlusion and resistance to erosive challenges

**DOI:** 10.1186/s12903-021-01977-3

**Published:** 2021-11-30

**Authors:** Xiaoyi Zhao, Lin Wang, Jie Pan, Hans Malmstrom, Yan-Fang Ren

**Affiliations:** 1 Department of General Dentistry, National Clinical Research Center for Oral Diseases, National Engineering Laboratory for Digital and Material Technology of Stomatology, Beijing Key Laboratory of Digital Stomatology, Peking University School and Hospital of Stomatology, Beijing, China; 2 University of Rochester Eastman Institute for Oral Health, 625 Elmwood Ave, Rochester, NY 14620 USA

**Keywords:** Dentin sensitivity, Dental erosion, Dentifrice, Dentin, Dentin tubules

## Abstract

**Background:**

Many studies have demonstrated efficacy of casein phosphopeptide (CPP) containing products for dentin tubule occlusion for treatment of dentin sensitivity, but their effectiveness under dynamic erosive challenges remains to be elucidated. The purpose of the present study was to investigate the effectiveness of a desensitizing dentifrice containing CPP in occluding dentin tubules and resisting erosive challenges in comparison to that containing polyvinyl methyl ether/maleic acid (PVM/MA) copolymers.

**Methods:**

A total of 33 dentin discs were prepared from coronal sections of human third molars and divided into 3 groups: a toothpaste containing CPP; a toothpaste containing PVM/MA and submicron silica; and a regular toothpaste (Controls). A soft-bristle toothbrush was used to brush the dentin discs with the dentifrices for 45 strokes in 30 s at a force of approximately 200 g. The brushing cycle was repeated after immersion of the dentin discs in artificial saliva overnight. The dentin discs were then challenged in orange juice for 10 min in an incubator rocking at 120 rpm. Three fields were randomly selected on each dentin disk surface to assess dentin tubule occlusions after each brushing cycle and after orange juice challenge with a 3D laser scanning microscope. Specimen cross sections were examined with a scanning electron microscope equipped with energy dispersive spectroscopy (SEM/EDS).

**Results:**

After the first and second cycles of brushing, dentin tubules were occluded on average by 56.3% and 85.7% in CPP group, 66.2% and 88.1% in PVM/MA group, and 0.0 and 13.0% in the controls, respectively. There were no statistically significant differences in dentin tubule occlusions between the CPP and PVM/MA groups after two cycles of brushing (p>0.05). After dynamic erosive challenges with orange juice, 20.3% of the dentin tubules in the CPP group, 79.1% in the PVM/MA group and none in the control remained occluded (P<0.05). SEM/EDS imaging showed that dentin tubules were blocked with plugs containing dentifrice substances in CPP and PVM/MA groups after treatments, but none in the controls.

**Conclusions:**

Desensitizing dentifrices containing CPP or PVM/MA could effectively occlude dentin tubules after two cycles of brushing. PVM/MA in combination with submicron silicon dioxide exhibited stronger resistance to dynamic erosive challenges by acidic beverages. Inorganic fillers that can enter dentin tubules and resist erosive challenges may be key for desensitizing dentifrices.

## Background

Dentin sensitivity (DS) is defined as transient sharp pain associated with thermal, mechanical, chemical and osmotic stimuli that cannot be attributed to any other dental diseases [1]. The prevalence of DS has been reported as high as 92.1% [2] with an average of 33.5% in the general population [3]. Teeth sensitivity also occurs in 54-55% of the patients following subgingival scaling and root planning [4].

DS is associated with exposed dentin tubules that connect the dental pulp with the oral environment [1]. The pathogenesis of DS is not entirely clear, but the most widely accepted mechanism is the hydrodynamic theory. When subjected to external stimuli, dentin tubule fluids moves rapidly under hydrodynamic pressure, and activates the sensory receptors located in the dental pulp, thus causing transient pain [5]. Several therapeutic strategies have been developed to treat DS based on the hydrodynamic theory, including physically occluding the exposed dentin tubules to form a barrier and isolate the tubule contents from the external stimulation, or chemically desensitizing the sensory nerves in the dental pulp and thus prevent the transmission of sensory impulses. Studies on blocking the pulpal nerve responses by potassium salt products produced inconsistent results and the effectiveness of potassium dentifrices remains to be substantiated [6, 7]. And most potassium formulations require several weeks to exhibit their desensitizing effects [8]. Dentin tubule occlusion by dentifrices, directly or indirectly, has been shown to be an effective treatment for dentin sensitivity [1, 9].

Desensitizing dentifrices that adopt the tubule occlusion strategy often contain inorganic solid fillers and organic polymers that have adhesive properties towards dentin. Together with mineral solids, organic polymers form a coating layer on the surface and occluding plugs inside dentine tubules through diffusion through the porous dentin, physical adsorption, and chemical bonding and electrostatic interactions [10]. The coating layer and intra-tubular plugs should adhere to the dentin and tubule walls and resist salivary enzymatic and acidic erosions for the intervals that at least span the two brushing periods to maintain a sustained effect of DS prevention [9].

Casein phosphopeptides (CPP) containing the cluster sequence -Ser(P)-Ser(P)-Ser(P)-Glu-Glu- have a significant ability to stabilize calcium, phosphate and fluoride ions at high concentrations as amorphous nanocomplexes that included CPP-amorphous calcium phosphate (CPP-ACP) or CPP-amorphous calcium fluorophosphate (CPP-ACFP) [11]. CPP was first introduced by Reynolds at 1992 with a focus on its anticariogenic activity [12]. CPP-ACP has in recent years been used as a desensitizing agent for treating DS [13] or preventing DS from prepared abutment teeth [14]. It has been shown that dentifrice containing CPP-ACP could block dentin tubules in vitro [15], and the resultant tubule occlusion appeared to be stable when subjected to static immersion challenges in grape juice for 5 min [16].

Polyvinyl methyl ether / maleic acid (PVM/MA) copolymer is a widely used bioadhesive molecule. Its tissue adhesion characters have been utilized in denture adhesives [17], anti-gingivitis or desensitizing mouthwashes [10, 18]. When combined with inorganic fillers, PVM/MA performed better in dentine tubule occlusion than products that contained either the fillers or the copolymers alone [19].

An ideal desensitizing agent should not only act immediately after the application, but also provide durable effects that last at least till the next application. Exposures to dynamic acidic challenges can influence the durability of desensitizing effects. Though some studies have demonstrated the potential efficacy and stability of CPP containing products for dentin tubule occlusion [16], the effectiveness of these products under dynamic erosive challenges that simulating the prolonged sipping of acidic beverages has not been investigated. No study has compared the relative effectiveness between CPP and PVM/MA dentifrices in their actions on dentin tubule occlusion and resistance to erosive challenges.

The purpose of this study was to compare the relative effectiveness of a dentifrice containing CPP to that containing PVM/MA copolymers in occluding dentin tubules and to examine the stability of the dentifrice plugs in the dentin tubules following dynamic erosive challenges in vitro. We hypothesized that CCP containing dentifrices may form stable barriers on dentin surfaces and inside dentin tubules and resist dynamic erosive challenges in comparison to dentifrices containing the PVM/MA copolymers.

## Methods

**Fig. 1:**
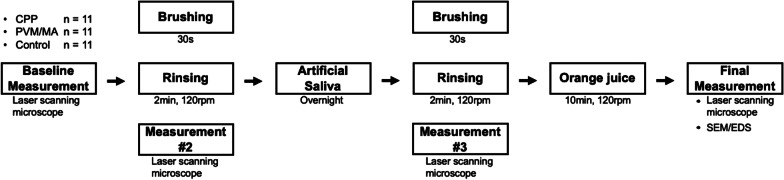
Flow diagram of the experimental design. CPP: casein phosphopeptide; PVM/MA: polyvinyl methyl ether/maleic acid; SEM: scanning electron microscopy; EDS: energy dispersive spectroscopy

The overall study design is illustrated with a flow diagram shown in Fig. 1. The details of the study procedures are described below.

### Sample collection and preparation

A total of 33 dentin discs, approximately 1.0 mm thick, were prepared from coronal sections of freshly extracted human third molars (within 30 days of extraction) as described elsewhere [9]. Tooth collection was conducted in accordance with the guidelines of Institutional Review Board of the authors’ institution (approval number: PKUSSIRB-202054044). The third molars were stored in 1% thymol solution immediately after extraction and then sterilized overnight in ethylene oxide before sample preparation. The occlusal part of the enamel was removed with a slow speed diamond saw to expose the dentin. A parallel cut was then made above the cementoenamel junction to produce a dentin disc that was approximately 2 mm in thickness. The dentin discs were ground on a 200-grit carbide plate to remove any remnants of the enamel on the occlusal side and the pulp horn on the pulp side of the disc, and polished with 400, 800 and 1200 grit carbide polishing papers [9]. The prepared dentin discs were cleaned for 3 min in an ultrasonic cleaner using 1% Micro-90 cleaner (International Product Corp., Burlington, NJ). The dentin discs were inspected under a 3D laser scanning microscope (VK-X200, Keyence, Osaka, Japan) to confirm that the dentin tubules were patent.

### Baseline sample measurements

An anti-acid nail polish was used to mark the dentin disc to identify the orientation landmarks for repeated imaging measurements of the samples before and after dentifrice treatments. 3D images of the dentin discs were obtained adjacent to the indentation marks using the 3D laser scanning microscope at magnifications of ×1000 and ×3000. Three different measurements were obtained on one sample. The low magnification (×1000) images were used for analysis of open and occluded tubules, and high magnification (×3000) images were used to measure the diameters of the dentine tubules. The degree of dentin tubule occlusion was calculated as percentage of tubules that were totally occluded in the examination fields. The diameter of the unoccluded or partially occluded tubules was measured as the longest dimension of the dentine tubule opening using an imaging software (Image J 1.53a, NIH, USA).

### Dentifrice treatments

Thirty-three dentin discs were randomly distributed into 3 groups, with 11 specimens per group. Ten specimens in each group were used for microscope measurement and one for SEM/EDS imaging of fractured cross-sections of the dentin discs. Group 1 (CCP group) was treated with dentifrice containing CPP and amorphous calcium and phosphate containing salts (Gobrite Desensitizing Paste, Beyond International Inc. Stafford, TX). Group 2 (PVM/MA group) was treated with dentifrice containing PVM/MA copolymer with submicron silica (0.2-0.3 microns) (Gobrite Whitening toothpaste Sensitivity Formula, Beyond International Inc. Stafford, TX). Group 3 was treated with a control dentifrice (Crest Regular Cavity Protection, Proctor & Gamble Inc., Cincinnati, OH, USA) (Table 1). Dentifrice slurries were prepared daily in a 1:3 ratio with artificial saliva by weight. A soft bristle toothbrush (purchased from the American Dental Association, Chicago, IL, USA) was used to manually brush the dentin disc using a circular motion with the dentifrice slurries for 45 strokes in 30 s at a force of approximately 200 g [9]. Before each brushing cycle, the brushing force was calibrated on a laboratory scale to ensure a consistent force of 200 g. After brushing, the discs were placed in deionized water and shaken in a rocking incubator at 37 ℃ for two minutes at a frequency of 120 rpm to simulate rinsing, and washed afterward for 5 s. The samples were then placed in deionized water for first post-treatment imaging as described in the baseline measurements.

**Table 1:** Compositions of dentifrices used in the current study

Group	Commercial name and manufacturer	Composition
CPP dentifrice	Beyond/Gobrite Desensitizing Paste (Beyond International Inc. Sugar Land, TX, USA)	Sodium Fluoride 0.32% (w/w), Casein Phosphopeptide, Sorbitol, Calcium Lactate, Dipotassium Phosphate, Hydrated silica (8 μm on average), Potassium Nitrate, Vegetable Glycerin, Calcium Nitrate, Xylitol, Sodium Hydroxide, Hydroxyethyl Cellulose, Titanium Dioxide, Hydroxyapatite, Cellulose Gum, Potassium Sorbate, Aroma, Sucralose, Thymol, Deionized Water,
PVM/MA dentifrice	Beyond/Gobrite Whitening Toothpaste Sensitivity Formula (Beyond International Inc. Sugar Land, TX, USA)	Sodium Fluoride 0.24% (w/w), PVM/MA Copolymer, Sorbitol, Titanium Dioxide, Potassium Nitrate, Silicon Dioxide (0.2 – 0.3 μm), Deionized Water, Propylene Glycol, PEG-1450, Sodium Lauryl Sulfate, Cellulose Gum, PEG-400, Hydroxyethyl Cellulose, Sodium Hydroxide, Aroma, Sodium Saccharin, Sodium Benzoate
Control dentifrice	Crest Regular Cavity Protection (Proctor & Gamble Inc., Cincinnati, OH, USA)	Sodium fluoride 0.243% (0.15% w/v Fluoride Ion), sorbitol, water, hydrated silica, sodium lauryl sulfate, trisodium phosphate, flavor, sodium phosphate, cellulose gum, sodium saccharin, carbomer 956, mica, titanium dioxide, Blue 1.

PEG, Polyethylene Glycol; PVM/MA, polyvinyl methyl ether/maleic acid

After the first post-treatment imaging, the samples were placed in 60 ml of artificial saliva overnight for 16 h till the following day, when the dentin discs were removed from the saliva and rinsed for 15 s in deionized water to prepare for the second cycle of brushing, which was processed the same as the first brushing cycle using the dentifrice slurries.

The artificial saliva contained the following chemicals in 1 L of distilled water: 0.33 g KH_2_PO_4_, 0.34 g Na_2_HPO_4_, 1.27 g KCl, 0.16 g NaSCN, 0.58 g NaCl, 0.17 g CaCl_2_, 0.16 g NH_4_Cl, 0.2 g urea, 0.03 g glucose, 0.002 g ascorbic acid [20]. Artificial saliva was freshly prepared and stored at 4 ℃.

### Post-treatment measurements

Imaging analysis as described in the baseline measurements were repeated after each cycle of dentifrice brushing treatments.

### Dynamic erosive challenges by orange juice

The dentin discs were removed from deionized water, immersed in 60 ml of orange juice (Minute Maid^TM^ Premium, pH of 4.11) evenly distributed in 6-well cell culture dishes for 10 min in an incubator rocking at 120 rpm to keep the solution well-mixed and simulate the fluid movement during the sipping of juice.

### Measurements after erosive challenges

The dentin discs were washed in deionized water for 15 s after orange juice erosion. Imaging analysis of the dentin discs was then performed for the fourth and final time as described in the baseline measurements.

### SEM/EDS imaging

The dentin samples for SEM/EDS imaging were dried in room temperature after treatment completion, fractured into two halves to expose the long axes of the dentin tubules and coated with gold. The dentin tubule plugs were then observed using a SEM (EVO18, Carl Zeiss NTS GmbH, Germany) and its chemical composition was measured with EDS using an XFlash X- ray spectrometer (XFlash 6130, Bruker Corporation, Billerica, USA) on board the SEM.

### Statistical analyses

Sample size estimation was based on previous studies using dentifrice for dentin tubule occlusion [9], and on our pilot test which showed that on average about 80% (SD=10%) of the tubules were occluded after a single brushing with the PVM/MA dentifrice. Based on this pilot data, we would need 9 samples per group to detect a 20% difference in mean tubule occlusions between the CPP group and the PVM/MA group with 90% power at an alpha level of 0.05. We decided to include 11 specimens per group. The proportions of occluded dentin tubules compared among the 3 groups after the first and second dentifrice treatments and erosive challenge cycles using contingency table tests. The diameters of dentine tubule openings before and after treatments in each group and in teeth exposed to orange juice were compared using analysis of variance and a post hoc Fisher’s LSD tests. All statistical analyses were based on 2-tailed tests at an alpha level of 0.05. Bonferroni corrections were applied to all statistical analyses to adjust for random errors associated with multiple comparisons.

## Results

**Fig. 2:**
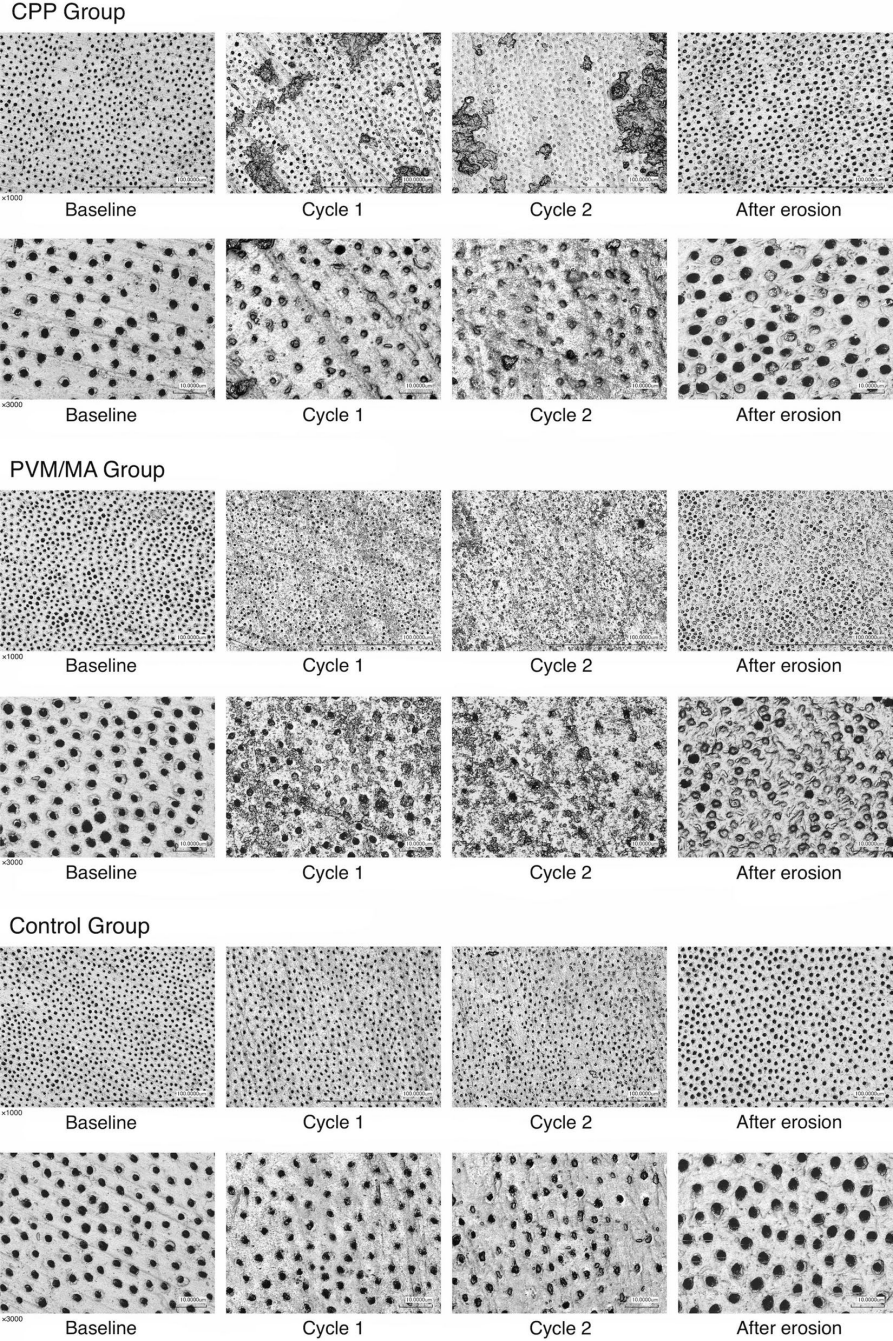
Dentin discs at baseline, after first and second brushing with the study dentifrices, and after dynamic erosive challenges by orange juice. Images were taken with 3D laser scanning microscope at magnifications of ×1000 and ×3000

As shown in Fig. 2, some discontinuous patches of deposits were observed on the dentin surfaces in CPP group after brushing with the toothpaste and rinsing. A more uniform particulate substances were seen on the dentin surfaces in PVM/MA group. No apparent deposits were observed on the dentin surfaces in the control group. Under high magnification, nearly all dentin tubules were patent at baseline before brushing. After the first and second cycles of brushing, dentin tubules were occluded on average by 56.31% and 85.66% in CPP group, by 66.15% and 88.07% in PVM/MA group, and by 0.0 and 12.96% in the controls, respectively (p<0.01) (Table 2). There were no statistically significant differences in dentin tubule occlusions between the CPP and PVM/MA groups after two cycles of brushing (*p*>0.05). After dynamic erosive challenges with orange juice, 20.28% of the dentin tubules in the CPP group, 79.08% in the PVM/MA group and 2.37% in the controls remained occluded (*p*<0.01) (Fig. 2).

**Table 2:** Occluded percentage (%) of dentin tubules following 2 cycles brushing and after erosion (mean ± SD)

	Baseline	Cycle 1 brushing	Cycle 2 brushing	After erosion
CPP group	0	56.31±15.23^a^	85.66±5.87^a^	20.28±10.68^a^
PVM/MA group	0	66.15±13.20^a^	88.07±5.27^a^	79.08±9.68^b^
Control group	0	0^b^	12.96±7.84^b^	2.37±4.38^c^
P	NS	<0.01	<0.01	<0.01

Different letters in groups in the same column denote statistically significant difference; when the same letter is present in different groups from the same column it denotes that these groups are not statistically different. CPP, casein phosphopeptide; PVM/MA, Polyvinyl methyl ether/ maleic acid; NS, not significant

Dentin tubules that were not fully occluded after brushing appeared to be smaller in diameter in the CPP (1.65±0.25) and PVM/MA (1.58±0.28) groups as compared to the controls (2.17±0.31) (*p*<0.05), indicating partial blockage of the tubule openings through deposits of dentifrice substances on the tubule walls (Fig. 2; Table 3). After erosive challenges with orange juices, dentin tubule openings increased in diameter in the control group (3.24±0.38) and became significantly larger than those in the CPP (2.40±0.38) and PVM/MA (2.08±0.26) groups (P<0.05). Dentifrice containing PVM/MA and submicron silica exhibited better anti-erosive effect as the tubule openings were smaller in this group than those in the CPP group (*p*<0.05) (Fig. 2; Table 3).

**Table 3:** Dentin openings diameter (µm) of dentin tubules following 2 cycles brushing and after erosion (mean ± SD)

	Baseline	Cycle 1 brushing				Cycle 2 brushing			After erosion
CPP group	2.33±0.19 ^Aa^	1.96±0.25^Ba^		1.65±0.25^Ca^				2.40±0.38^Aa^	
PVM/MA group	2.24±0.34 ^Aa^	1.87±0.16^Ba^		1.58±0.28^Ca^				2.08±0.26^Ab^	
Control group	2.19±0.16 ^Aa^	2.14±0.14^Ab^		2.17±0.31^Ab^				3.24±0.38^Bc^	
P	NS	<0.05		<0.05				<0.05	

Different letters in groups in the same column denote statistically significant difference; when the same letter is present in different groups from the same column it denotes that these groups are not statistically different. Lower letters to the column and capital letters to the line. CPP, casein phosphopeptide; PVM/MA, Polyvinyl methyl ether/ maleic acid; NS, not significant

**Fig. 3:**
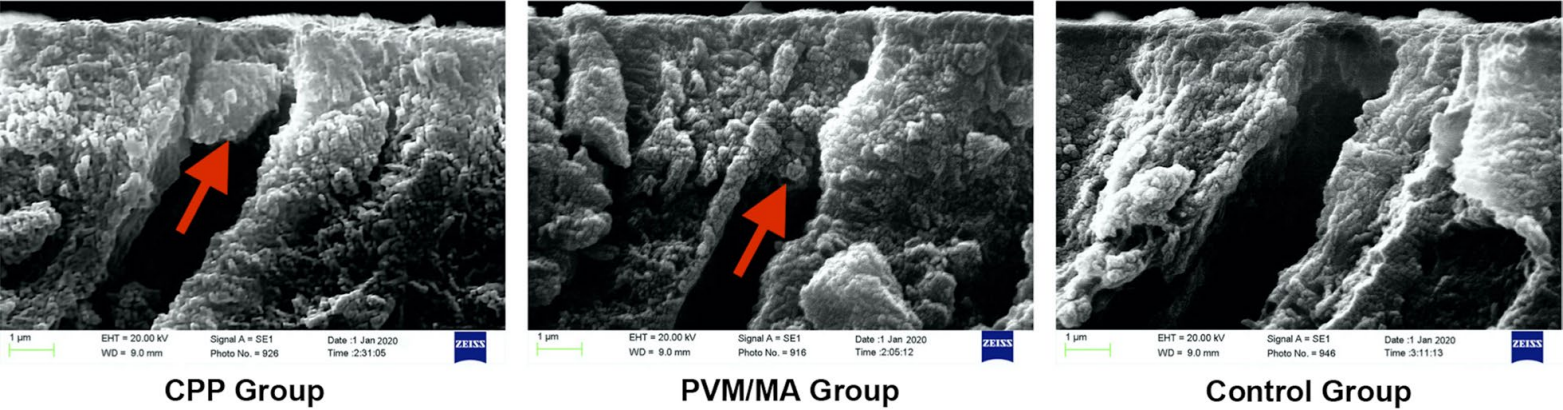
Scanning electron microscopic images of dentin discs showing tubule openings occluded by dentifrice plugs (arrows) after dynamic erosive challenges by orange juice. Dentin tubule plugs were present in the CPP and PVM/MA groups but not in the control group

**Fig. 4:**
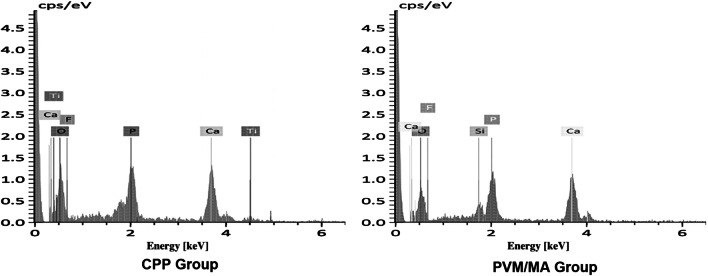
Energy dispersive X-ray spectroscopy shows chemical composition of dentin tubule plugs in the CPP and PVM/MA groups. Titanium element was present in CPP dentifrice as titanium dioxide. Silica element was present in the PVM/MA dentifrice as silicon dioxide

As shown in Fig. 3, dentin tubule plugs 1–2 μm in length were formed in the CPP and PVM/MA groups but not in the control group (Fig. 3). EDS confirmed that the substances that occluded the dentin tubules in the CPP and PVM/MA group were consistent with the components of the study dentifrices (Fig. 4).

## Discussion

The findings of the present study indicate that the dentifrice containing CPP can form intra-tubular plugs and occlude the dentin tubule openings comparable to the dentifrice containing PVM/MA and submicron silica fillers, and provide some protection to exposed dentin surfaces against erosive challenges by orange juice. A majority of the intratubular plugs remained in the PVM/MA group and provided better protection against erosion than in the CPP group after aggressive erosive challenges, which suggests that the adhesive properties of the organic polymers may play an important role in desensitizing dentifrices aimed at blocking dentin tubules.

Bioadhesive polymers are often used for controlling release of medical and dental therapeutic agents. PVM/MA copolymer has been widely used in dentistry [17, 21–23], and its bioadhesive properties are related to the formation of carboxylic groups from the polyanhydride residues of the copolymer [24]. It has been shown that dentifrice containing PVM/MA copolymers could improve the retention of fluoride after a single brushing application compared to those without bioadhesive copolymers[19]. PVM/MA could bind to glycoprotein through mechanical interpenetration and weak chemical bonds [25, 26]. It has also been shown that PVM/MA molecules readily bind to type I collagen that are present on exposed dentin surfaces, likely through weak chemical bonds including hydrogen bonds and van der Waals force [27]. A thin layer of dentifrice substances remained on dentin surfaces after application when PVM/MA was included in the formulae, which served as a barrier against erosive challenges. The combination of PVM/MA copolymers with submicron inorganic fillers facilitated dentine tubule occlusions and played an important role on erosion prevention in the early stage of erosive challenge cycles as compared to the CPP product. These findings are consistent with our previous study that showed dentifrice plugs formed by PVM/MA and silica were stable in dentin tubules after challenges by saliva, rinsing and orange juice, which signified that the intra-tubular plugs adhered well to the dentin tubule walls and were resistant to salivary enzymatic and acidic erosions[9].

CPP has a positive role on the enhancement of calcium, phosphate and fluoride solubility and their stabilization on tooth surfaces and prevents tooth demineralization[12]. The calcium, phosphate and fluoride ions are provided from calcium lactate, dipotassium phosphate and sodium fluoride in the current dentifrice formulation. Fluoride ions are designed to be incorporated into the amorphous calcium phosphate phase to form an amorphous calcium fluorophosphate (ACFP) complex that could be stabilized by CPP. The CPP-ACFP complex has been shown to have excellent remineralization efficacies owing to the synergistic effect between CPP, calcium, phosphate and fluoride ions[28, 29]. It has a broad spectrum of bioactivities including promotion of remineralization, prevention of demineralization, and protection against erosion and DS [30]. However, it may take up to several days to 4 weeks of application for CPP products to show its effect in remineralization [28, 31, 32], which indicates that it is vitally important for these products to adhere to tooth surfaces and withstand erosive challenges.

A previous study showed that a CPP containing dentifrice blocked 43.2% of the dentin tubules after one application [15]. We found that repeated application may increase the proportion of blocked dentin tubules to greater than 80%, and that the diameters of tubule openings became smaller even they were not totally blocked. The blockage plugs were relatively short (1-2 μm) and confirmed to be dentifrices substances by EDS. However, these plugs were not as stable as those formed in the PVM/MA group and largely disappeared after dynamic erosive challenges by orange juice. Therefore, its effectiveness against DS is at best doubtful if the dentin tubule plugs could be easily removed by dietary acid challenges [33].

In addition to the organic CPP or PVM/MA molecules, the inorganic fillers of the study dentifrices may have played important roles in tubule blockage and tubule plug stabilities against dynamic erosive challenges. In the CPP group, the inorganic fillers are hydrated silica on average 8 μm in diameters, which are too large to enter dentin tubules that are 2 to 3 μm in diameters. In the PVM/MA group, however, the inorganic fillers are primarily comprised of submicron silicon dioxide (0.2-0.3 microns) particles, which may easily enter the open dentin tubules and form more stable plugs together with the adhesive polymers.

DS is often associated with dental erosion [34]. Erosive challenges on exposed dentin surfaces may widen the openings of the dentin tubules and exacerbate the DS. Continued acidic challenges will induce progressive deteriorations of dental hard tissues [20]. The findings of the present study using 3D laser scanning microscope showed that there is a deposit layer of dentifrice substances in the tubules that were not completely blocked in both the CPP and PVM/MA groups. This protective layer may reduce the detrimental effects of acidic erosive challenges. The diameters of the tubule opening increased by nearly 50% from 2.19 to 3.24 μm on average after orange juice challenges in the control group, but those in the CPP and PVM/MA groups remained relatively stable. These findings suggest that these dentifrices may have potential protective effects against dental erosion.

## Conclusions

Within the limitations of this in vitro study, we conclude that both the CPP and PVM/MA dentifrices may provide some protective effects to exposed dentin with open dentin tubules. The dentifrice containing PVM/MA with submicron silica fillers occluded more dentin tubules and promoted less dentin alteration after erosive challenges in comparison to the CPP and control group.

## Data Availability

Available upon request from the corresponding author.

## Abbreviations

CPP: Casein phosphopeptide
PVM/MA: Polyvinyl methyl ether/maleic acid
SEM: Scanning electron microscope
DS: Dentin sensitivity
CPP-ACP: CPP-amorphous calcium phosphate
CPP-ACFP: CPP-amorphous calcium fluorophosphate
EDS: Energy dispersive spectroscopy
